# miRmap web: comprehensive microRNA target prediction online

**DOI:** 10.1093/nar/gkt430

**Published:** 2013-05-28

**Authors:** Charles E. Vejnar, Matthias Blum, Evgeny M. Zdobnov

**Affiliations:** ^1^Department of Genetic Medicine and Development, University of Geneva Medical School, Geneva, Switzerland, ^2^Swiss Institute of Bioinformatics, Geneva, Switzerland and ^3^Imperial College London, South Kensington Campus, London, UK

## Abstract

MicroRNAs (miRNAs) posttranscriptionally repress the expression of protein-coding genes. Based on the partial complementarity between miRNA and messenger RNA pairs with a mandatory so-called ‘seed’ sequence, many thousands of potential targets can be identified. Our open-source software library, miRmap, ranks these potential targets with a biologically meaningful criterion, the repression strength. MiRmap combines thermodynamic, evolutionary, probabilistic and sequence-based features, which cover features from TargetScan, PITA, PACMIT and miRanda. Our miRmap web application offers a user-friendly and feature-rich resource for browsing precomputed miRNA target predictions for model organisms, as well as for predicting and ranking targets for user-submitted sequences. MiRmap web integrates sorting, filtering and exporting of results from multiple queries, as well as providing programmatic access, and is available at http://mirmap.ezlab.org.

## INTRODUCTION

MicroRNAs (miRNAs) are short (∼22 nt) noncoding RNAs that guide the RNA-induced silencing complex (RISC) to posttranscriptionally repress the expression of protein-coding genes by binding to targeted messenger RNAs (mRNAs) (1–3). While the detailed mechanism of this guidance is not yet resolved, binding between the mRNA and the miRNA from position 2–7 (or 8) from the 5′ end usually triggers RISC-mediated repression (4). In the miRNA–mRNA binding map based on Ago HITS-CLIP (HIgh-Throughput Sequencing of RNA isolated by CrossLinking ImmunoPrecipitation) (5), 73% of the studied target sites have a seed match. While searching for seed matches in 3′-UTR is a widely used feature for miRNA target prediction, it yields a very high number of potential targets. For example, human 3′-UTRs have 9.5 million potential targets defined as 7-mer seed matches of all human miRNAs, which means that on average a miRNA potentially targets ∼25% of the human genes. While this estimation is probably an upper bound, and as in a specific cell or tissue, not all miRNAs and all genes are expressed, thereby reducing the number of potential targets, prioritization of predictions is necessary to experimentally test the most probable targets first. We recently proposed a ranking function of miRNA-mediated repression strength parameterized from experimentally measured effects on mRNA or protein levels resulting in prediction of biologically meaningful potential targets (6).

**Figure 1.**
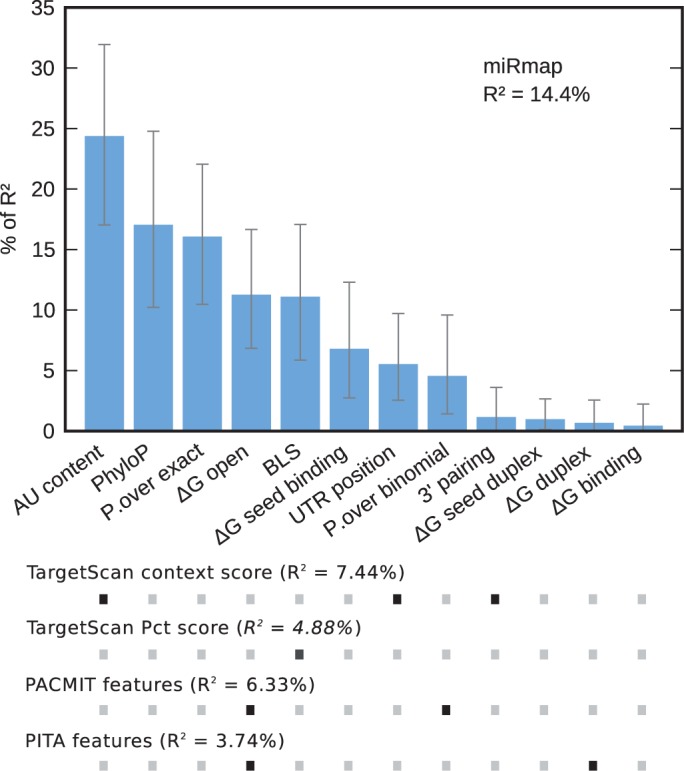
Feature relative importance and performance of the miRmap model (as R^2^) compared with similar miRNA target predictions software. Similar features of each software to miRmap are marked with a black dot, with their performance also evaluated as R^2^. R^2^ is the proportion of variance explained by the model on transcriptomics data (6). TargetScan conservation score (Pct, probability of conserved targeting) (7) R^2^ was computed directly with TargetScan predictions, as Pct, which is based on the BLS feature (marked as a dark gray dot), is not included in miRmap.

Implemented as an open-source Python software library, the miRmap library implements a comprehensive range of features using thermodynamic, probabilistic, evolutionary and sequence-based approaches to rank potential miRNA targets using information beyond the seed match. We compared (6) the power of individual approaches to predict the repression strength of miRNA–mRNA pairs, assessed using data from transcriptomics, immunopurification, proteomics and polysome fractionation high-throughput experiments, and combined the features with a linear model to predict the miRNA repression strength as the ‘miRmap score’. This score doubles the performance of the TargetScan context score measured as the proportion of explained variance of a transcriptomics data set (6). Moreover, our comprehensive set of features covers features included in many other tools (Figure 1). For example, PITA (8) ranks targets only with ‘ΔG total’, and PACMIT (9) uses a combination of ‘ΔG open’ and ‘P.over binomial’. As the miRmap score is more predictive than these individual features (6), miRmap can be used as a reference tool. Here, we present ‘miRmap web’, a web application based on a public REST (REpresentational State Transfer) service. With miRmap web, biologists can easily, through a user-friendly and feature-rich graphical interface, browse precomputed miRmap predictions from model organisms, and also predict and rank miRNA targets on their own sequences.

## IMPLEMENTATION

### miRmap features and model

Thermodynamic features of miRmap, which we described previously (6), include ‘ΔG duplex’ and ‘ΔG binding’ that evaluate the energy of the miRNA–mRNA duplex, ‘ΔG open’ that evaluates the energy necessary to unfold the 3′-UTR and make the area accessible for RISC binding and ‘ΔG total’ [also named ‘ΔΔG’ by Kertesz et al. (8)] that sum ‘ΔG duplex’ and ‘ΔG open’. Since the publication of the miRmap library, we have added new features, ‘ΔG seed duplex’ and ‘ΔG seed binding’, that specifically evaluate the seed region (10,12). Our model (6) explains 12.7% (R^2^) of variance on the Grimson et al. data set (13), whereas the TargetScan context score (‘AU content’, ‘3′ pairing’ and ‘UTR position’) explains 7.49% with the same type of linear model (6). With the addition of the ‘ΔG seed duplex’ and ‘ΔG seed binding’ features, our model now explains 14.4% of the variance (Figure 1). It is worth nothing that TargetScan predictions also include an independent filter on conservation (7), named Pct. This score, based on the ‘Branch Length Score (BLS)’ feature, explains 4.88% of the variance. As described in our previous publication (6), fold-change distribution confirmed greater enrichment of stronger experimental repression with greater miRmap score (Supplementary Figure S1). This analysis was also applied specifically to 6-mer seeds and also showed increased performance (Supplementary Figure S2). The sequence-based features are, as described by the TargetScan context score (13), as follows: ‘AU content’ that is a weighted count of A and U nucleotides around the seed match, ‘UTR position’ that takes into account the enhanced repression for sites that are close to one end of the 3′-UTR and ‘3′ pairing’ that evaluates the contribution of the pairing on the opposite side to the miRNA seed. As only limited regions of 3′-UTRs have regulatory or structural roles, we introduced features in miRmap with probabilistic and evolutionary points of view. In the probabilistic approach, we modeled the 3′-UTR sequence composition with a Markov process (order 1) and determined the expected probability of finding at least *n* occurrences of the seed match, either with a binomial approximation (‘P.over binomial’) or with an exact solution (‘P.over exact’). The evolutionary approach assesses the conservation of the target sites compared with the rest of the 3′-UTR as measured by the ‘BLS’ and ‘PhyloP’ features. The ‘BLS’ evaluates the evolutionary time during which the seed match was present in a given set of species (14), while the ‘PhyloP’ feature detects negative selection pressure on the target sites within a statistical framework (11). MiRmap brings together common features of miRNA target prediction and extends these with novel features including ‘ΔG binding’, ‘ΔG seed binding’, ‘P.over exact’ and ‘phyloP’ to provide the first truly comprehensive open-source library for target predictions.

### Precomputed predictions

As well as allowing predictions for user-submitted sequences, miRmap includes predictions for the annotated miRNAs [miRBase 19 (15)] and genes [Ensembl 69 (16)] of eight species: Human, Chimpanzee, Mouse, Rat, Cow, Chicken, Zebrafish and Opossum.

### Web server implementation

As our service allows both interrogating a database and computing predictions, we developed a framework that ensures a reliable and responsive service. All computations on the server side are performed asynchronously: multiple users can search and compute predictions at the same time without impacting other users, with our web server computing capability as the only limit. This implementation was written using the Tornado framework (tornadoweb.org) and deployed using Nginx (nginx.org) reverse proxy.

### Client implementation

MiRmap web can be used as a web application or as a REST service. The web application is a single web page for ‘click&play’ usage integrating the form for the user query and the dynamically updated results table. Users can also easily retrieve prediction data sets or predict targets in a more programmatic way using the REST service. We used the widely used ExtJS Javascript toolkit (sencha.com) to build our web application and obtain a homogeneous look and feel as well as flexible configurability. A detailed documentation of the REST service, as well as examples, is included in help section of miRmap Web site.

## USAGE

### Input

**Figure 2.**
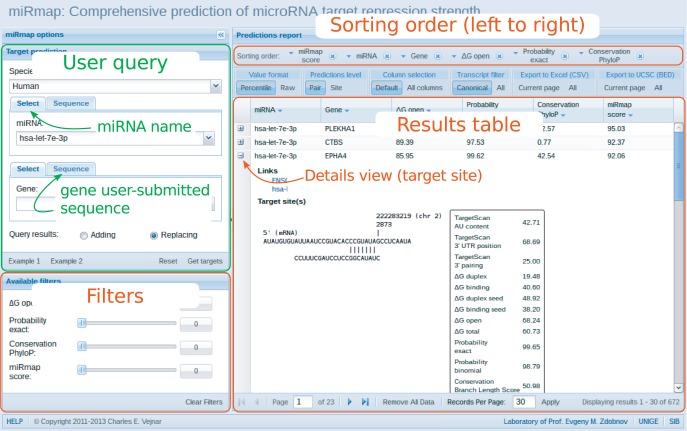
miRmap web application page describing the integration of the user query, results table with filtering and sorting parts.

MiRmap web includes facilities for both browsing precomputed miRNA target predictions, and for online computing of miRNA targets on user-submitted sequences. The first step is to select a species (Figure 2). The user may then ‘Select’ or input the ‘Sequence’ of a miRNA and/or a protein-coding gene. It is important to note that for browsing precomputed predictions, users may select a miRNA or a gene, or both, with an auto-complete capability to facilitate rapid selections The gene selection and sequence input options may be combined such that the user may input a miRNA sequence and select a gene/transcript by name, or *vice versa*. When a gene name or identifier is entered, the canonical transcript of that gene is selected; it is also possible to select a specific transcript by directly entering the transcript identifier. When the form is valid, the user may then submit his/her query by clicking on the ‘Get targets’ button.

## RESULTS

Results from user-submitted queries return feature values for each miRNA–mRNA pair by default (the ‘Pair’ view), where each record is expandable to access additional details about each predicted target site, or for each target site by selecting the ‘Site’ view. By default, only a selection of the complete set of all miRmap features is displayed, and intuitive options from the toolbar allow users to display all features or customize their feature selections. Additional toolbar options enable (i) switching between ‘Percentile’ and ‘Raw’ score formats, (ii) multicolumn ordered sorting and (iii) flexible filtering of the results table to suit the user’s requirements. Raw miRmap scores for each feature, e.g. ‘ΔG duplex’ in kcal/mol and ‘P.over exact’ as a probability, can be converted to percentiles to simplify and standardize a scale from strong to weak repression. This is achieved by ranking targets for each species from the weakest to the strongest predicted repression for each individual feature and assigning scores to percentiles from 0 to 100%, with 100 representing the strongest repression. Users therefore do not have to be familiar with the ranges of different feature values, nor their directions, i.e. whether larger or smaller values correspond to higher or lower repression. Results are sorted by default according to their miRmap scores, but users may define their own sorting functions on each selected feature through a simple ‘drag&drop’ ordering of each column in the results table. In addition, filters may be applied to limit results to only canonical transcripts for each gene as well as by setting minimum or maximum scores for each feature using sliding selectors. These customized results tables may then be easily exported as CSV (comma-separated values) formatted files and saved for viewing in common spreadsheet applications such as Excel, or as BED files for viewing in the UCSC (17) genome browser.

## CONCLUSIONS

With this integrated web interface, featuring easy filtering and sorting options, miRmap web aims to provide biologists interested in a particular miRNA or gene the means to prioritize their research with confident ranking of miRNA targets using the miRmap model. In contrast to most other miRNA target prediction web interfaces, miRmap web covers a wide range of possible usage by providing both precomputed and online predictions. Updated 3′-UTR annotations and newly discovered miRNAs can therefore be easily computationally tested with a state-of-the-art analytical tool.

## SUPPLEMENTARY DATA

Supplementary Data are available at NAR Online: Supplementary Figures 1–2.

## References

[1] Bartel DP (2009). MicroRNAs: target recognition and regulatory functions. Cell.

[2] Muljo SA, Kanellopoulou C, Aravind L (2010). MicroRNA targeting in mammalian genomes: genes and mechanisms. Wiley Interdiscip. Rev. Syst. Biol. Med..

[3] Axtell MJ, Westholm JO, Lai EC (2011). Vive la différence: biogenesis and evolution of microRNAs in plants and animals. Genome Biol..

[4] Brennecke J, Stark A, Russell RB, Cohen SM (2005). Principles of microRNA-target recognition. PLoS Biol..

[5] Chi SW, Zang JB, Mele A, Darnell RB (2009). Argonaute HITS-CLIP decodes microRNA-mRNA interaction maps. Nature.

[6] Vejnar CE, Zdobnov EM (2012). miRmap: comprehensive prediction of microRNA target repression strength. Nucleic Acids Res..

[7] Friedman RC, Farh KK, Burge CB, Bartel DP (2009). Most mammalian mRNAs are conserved targets of microRNAs. Genome Res..

[8] Kertesz M, Iovino N, Unnerstall U, Gaul U, Segal E (2007). The role of site accessibility in microRNA target recognition. Nat. Genet..

[9] Marín RM, Vanícek J (2011). Efficient use of accessibility in microRNA target prediction. Nucleic Acids Res..

[10] Garcia DM, Baek D, Shin C, Bell GW, Grimson A, Bartel DP (2011). Weak seed-pairing stability and high target-site abundance decrease the proficiency of lsy-6 and other microRNAs. Nat. Struct. Mol. Biol..

[11] Pollard KS, Hubisz MJ, Rosenbloom KR, Siepel A (2010). Detection of nonneutral substitution rates on mammalian phylogenies. Genome Res..

[12] Hausser J, Landthaler M, Jaskiewicz L, Gaidatzis D, Zavolan M (2009). Relative contribution of sequence and structure features to the mRNA binding of Argonaute/EIF2C-miRNA complexes and the degradation of miRNA targets. Genome Res..

[13] Grimson A, Farh KK, Johnston WK, Garrett-Engele P, Lim LP, Bartel DP (2007). MicroRNA targeting specificity in mammals: determinants beyond seed pairing. Mol. Cell.

[14] Stark A, Lin MF, Kheradpour P, Pedersen JS, Parts L, Carlson JW, Crosby MA, Rasmussen MD, Roy S, Deoras AN (2007). Discovery of functional elements in 12 drosophila genomes using evolutionary signatures. Nature.

[15] Kozomara A, Griffiths-Jones S (2011). miRBase: integrating microRNA annotation and deep-sequencing data. Nucleic Acids Res..

[16] Flicek P, Amode MR, Barrell D, Beal K, Brent S, Carvalho-Silva D, Clapham P, Coates G, Fairley S, Fitzgerald S (2012). Ensembl 2012. Nucleic Acids Res..

[17] Fujita PA, Rhead B, Zweig AS, Hinrichs AS, Karolchik D, Cline MS, Goldman M, Barber GP, Clawson H, Coelho A (2011). The UCSC genome browser database: update 2011. Nucleic Acids Res..

